# Acceptability of virtual reality for training health professions students in serious illness communication: A cross-sectional study with educators^☆^

**DOI:** 10.1016/j.pecinn.2025.100411

**Published:** 2025-06-24

**Authors:** Aleksandrina Skvortsova, Stephanie Stiel, Kambiz Afshar, Hanna A.A. Röwer, Claudia Bausewein, Irene Hartigan, Mohamad M. Saab, Sandra Martins Pereira, Pablo Hernández-Marrero, Jan Hrdlička, Jiri Wild, Kateřina Rusinová, Martin Loučka, Lucie Hrdličková, Martin Zielina, Cathy Payne, Liesbeth M. Van Vliet

**Affiliations:** a Leiden University, The Netherlands; b Institute for General Practice and Palliative Care, Hannover Medical School, Germany; c LMU University Hospital Munich, Munich, Germany; d Catherine McAuley School of Nursing and Midwifery, University College Cork, Cork, Ireland; e Universidade dos Açores | Fundação Gaspar Frutuoso, CEEAplA: Center of Applied Economic Studies of the Atlantic, Ponta Delgada, São Miguel Island, Azores, Portugal; f ComGuide, Prague, Czech Republic; g First Faculty of Medicine, Charles University, Prague, Czech Republic; h Third Faculty of Medicine, Charles University, Prague, Czech Republic; i Second Faculty of Medicine, Charles University, Prague, Czech Republic; j European Association for Palliative Care, Vilvoorde, Belgium

**Keywords:** Communication, Serious illness, Virtual reality, Health education, International online surveys

## Abstract

**Objectives:**

This study investigates i) acceptability, ii) predictors of intention to use, iii) barriers and facilitators, and iv) perceived advantages and disadvantages of incorporating virtual reality (VR) into serious illness communication training from the perspective of health professions educators in Europe.

**Methods:**

An online survey was distributed using snowball sampling across health professions educators involved in the creation and/or delivery of difficult communication courses (as educators, developers, coordinators).

**Results:**

Seventy-five educators from 11 European countries involved in teaching serious illness communication skills completed the survey. While educators viewed VR positively and saw it as useful, their intention to implement it was moderate, possibly, due to low compatibility with current teaching methods and social norms. Major barriers reported by participants included financial constraints (62.7 %) and lack of VR training (54.7 %), while key facilitators were training availability (22 %) and technical support (11 %).

**Conclusion/innovation:**

Educators perceive VR as a potential supplemental tool in difficult communication education; however, overcoming financial, training, and integration barriers is essential for its broader adoption and curricular integration. Further research is necessary to validate VR's effectiveness in developing the nuanced communication skills critical for serious illness communication.

**Innovation:**

VR technology is a promising innovative tool for medical communication training.

## Highlights

• Virtual reality has potential to support teaching of serious illness communication.
• Educators had a generally positive attitude toward VR's usefulness.
• Sufficient funding, training opportunities and technical support are necessary.
• The lack of human interaction is frequently mentioned as a disadvantage.
• Immersive nature of VR is frequently mentioned as an advantage.

## 1 Introduction

When caring for seriously ill patients, communication (e.g., delivering bad news about a diagnosis or prognosis) is part of everyday work of healthcare professionals. The quality of these conversations not only shapes patients' understanding of their condition and prognosis but also impacts their quality of life and treatment decisions [[1], [2], [3]]. However, having serious illness conversations is a significant source of stress for healthcare professionals that can affect their long-term mental health and lead to burnout [4,5]. It has been shown that healthcare professionals who feel insufficiently trained in communication report higher levels of burnout compared to those who feel adequately trained [6].

Healthcare professionals must employ various communication strategies to achieve effectiveness in serious illness communication. Certain communication techniques, like open information-disclosure, verifying patients' understanding through teach-back [7,8], and expressing empathy, including reassurance, positively impact patient outcomes, such as disease insight, emotional well-being, and information retention [[9], [10], [11], [12], [13]]. At the same time, often health profession education prioritises biomedical knowledge over psychosocial skills, leading to a lack of these communication competencies required for difficult conversations among many healthcare professionals [[14], [15], [16]].

Existing serious illness communication trainings (e.g., Vitaltalk and the Serious Illness Care Programme) provide practical strategies and utilize role-played model simulations; however, they do not, to the best of our knowledge, incorporate immersive technologies like VR. VR is a powerful modality of simulation, offering immersive, realistic environments that enhance experiential learning and skill development in complex scenarios. Systematic reviews and meta-analyses on VR in healthcare education have shown its effectiveness in improving learner engagement, knowledge retention, and skill acquisition, highlighting VR's potential as a valuable supplement to traditional training methods [[18], [19], [20], [21]]. With the rapid advancements in VR and artificial intelligence, it is now possible to create realistic virtual environments where students can practice a wide range of communication skills by interacting with simulated patients in diverse and challenging scenarios. Such patients can be programmed to represent diverse age groups, genders, and ethnicities, various disease trajectories and disease situations, as well as to exhibit various reactions. This provides students with the opportunity to refine their communication skills across a broad spectrum of scenarios [17]. Research shows that VR training has advantages over regular training methods, such as being more engaging and cost-effective [18,22,23]. To date, a number of studies demonstrated that it is possible to successfully train medical communication skills using various types of virtual environments [24,25], and VR in some cases might be even more effective than role-play in training empathic responses in healthcare providers [26]. It remains unknown how health professions educators feel about accepting and implementing VR technology for training health professions students in serious illness communication. Therefore, the overall aim of this study is to investigate the views of health professions educators on using VR for training healthcare professions students in serious illness communication in Europe. The rationale for focusing on educators' perspectives specifically, as educators play a critical role in integrating VR into curricula. The objectives of this study are four-fold: (i) to investigate the acceptability of using VR technology in difficult communication education from the perspective of health professions educators; (ii) to assess what predicts the intention to use VR by health professions educators; (iii) to explore perceived barriers and facilitators for using VR; (iv) to identify advantages and disadvantages of using VR in serious illness communication education.

## 2 Methods

### 2.1 Design

This is a cross-sectional exploratory online survey. This type of studies is effective in providing a snapshot of the current behaviours, attitudes, and perspectives of participants about the topic being studied, particularly in the field of education research [27].

### 2.2 Participants and recruitment

Study participants were European health professions educators involved in the creation and/or delivery of courses on communication in serious illness for healthcare students (as educators, developers/ instructional designers, and/or coordinators), and all demonstrated proficiency in English. An advertisement poster for the survey was widely disseminated through the contacts of the researchers from the seven universities represented in the research team as well as social media and newsletters (e.g., from Associations of Medical Education, European Association for Palliative Care). Participants across the whole world were able to fill in the survey; however, only data from participants in Europe is presented in the current manuscript. Data collection took place between November 2023 and March 2024. Before commencing with the survey, participants were given an information letter to read with the details of the study and emphasizing the voluntary nature of participating and were asked to sign an informed consent form (collected via Qualtrics).

### 2.3 Survey

At the start of the survey, participants read a short introduction to the concept of using VR for communication training. Participants were asked to answer the questions while having VR for training their students' communication within serious illness in mind. Questions were organised according to the following four dimensions: demographic variables; acceptability of using VR in serious illness communication; barriers and facilitators; and advantages and disadvantages.

#### 2.3.1 Demographics

The survey included several demographics and general questions, including country, sex, age, work experience, and role in the course on serious illness communication.

#### 2.3.2 Acceptability

To assess the acceptability of using VR in serious illness communication courses, we used an adapted version of the Assessing Determinants of Prospective Uptake of Virtual Reality (ADOPT-VR) questionnaire [28,29]. For the adaptation, we minimally adjusted the original questions from healthcare [28] to a health professions education context. For example, the question “I like the idea of using virtual reality with my clients” was replaced with “I like the idea of using virtual reality with my students”. ADOPT-VR is based on the Decomposed Theory of Planned Behaviour [30] and examines 10 constructs that constitute the acceptability of a new VR technology: attitude, perceived usefulness, ease of use, compatibility, social norms, peer influence, superior influence, perceived behavioural control, self-efficacy, facilitating conditions, and behavioural intention to use the technology. Each of these constructs constitutes a separate scale and higher scores on the scales indicate higher acceptability in the respective category. The ADOPT-VR has 24 items scored on a nine-point Likert scale (1: Strongly disagree – 9: Strongly agree) as well as 5 multiple-response and short-answer questions.

#### 2.3.3 Barriers and facilitators

To assess barriers, we used a check-box assessing barriers. We used answers, such as “Lack of time to learn how to use the virtual reality system” or “Lack of financial support” and included the following open-ended question: “The most significant barrier to my use of virtual reality is…” from the ADOPT-VR questionnaire. To assess facilitators, we used the open-ended question “What will help you to incorporate virtual reality into your teaching practice?” from the ADOPT-VR questionnaire.

#### 2.3.4 Advantages and disadvantages

To assess VR's advantages in serious illness communication courses, we asked a researcher-designed question: “In comparison to current teaching methods (e.g., face-to-face, simulation, blended-learning, clinical skills): "What do you feel are the advantages of using virtual reality in medical education on communication in serious illness?”. To assess disadvantages, we asked a self-created question: “What do you feel are disadvantages of using virtual reality in medical education on communication in serious illness?”

### 2.4 Analysis

Statistical analysis was performed in SPSS (IBM Corp. Released 2023. IBM SPSS Statistics for Windows, Version 29.0.2.0 Armonk, NY: IBM Corp). For quantitative analyses, statistical significance was set at *p* < .05. First, descriptive statistics of demographics were calculated. The categorical variables (country, gender, teaching role) were presented as absolute numbers and proportions. Continuous variables were presented as means and standard deviations.

Second, descriptive statistics for acceptability were calculated: the ADOPT-VR scores for each of the constructs measured by the questionnaire separately (attitude, perceived usefulness, perceived ease of use, compatibility, social norms, behavioural control, self-efficacy, time, technical support as well as intention to use VR in teaching practice) were calculated. The scores were presented as means and standard deviations, as they were normally distributed. Since ADOPT-VR does not have any threshold scores and guidelines for the interpretation of the scores, we a priori defined the levels for each of the scales by splitting the scales into three equal intervals. For the 3–27 scales (attitude, perceived usefulness, perceived ease of use, intention to use), the levels were defined as: low: 3–11; moderate: 12–19; high: 20–27. For the 2–18 scales (behavioural control, compatibility): low: 2–7; moderate: 8–13; high: 14–18. For the 5–45 scale (social norms): low: 5–18; moderate: 19–32; high: 33–45. For the 1–9 scales (technical support, time, self-efficacy): low: 1–3; moderate: 4–6; high: 7–9. To evaluate the reliability of the scales, the Chronbach's alpha was calculated for all scales of ADOPT-VR, except for self-efficacy, time and availability of tech support that include only one item.

Third, to assess the relationship between the constructs of the Decomposed Theory of Planned Behaviour (attitude, perceived usefulness, perceived ease of use, compatibility, social norms, behavioural control, self-efficacy, time and technical support) and intention to use VR in teaching communication in serious illness, a multiple regression analysis was performed with intention to use as a dependent variable and 9 constructs as predictors.

Fourth, the barriers and facilitators lists were created based on descriptives and classifying of the open-ended questions into categories. Lastly, advantages and disadvantages were created by classifying of the open-ended questions into categories. The categorization of responses was conducted by AS and reviewed by LV. Any discrepancies between their classifications were resolved through discussion.

### 2.5 Ethics

The study received ethical approval from the local research ethics committee (CEP, Institute of Psychology, Leiden University, NL; reference number: 2023-11-21-A. Skvortsova-V1–5097).

## 3 Results

### 3.1 Participants

In total, 79 educators completed the survey. Of those, four were from outside Europe, and their responses were not eligible for inclusion. Participants' characteristics (*n* = 75) are presented in Table 1.

**Table 1:** The characteristics of participants (n = 75).

Variables	Total (n = 75)
**Country**	
Germany	22 (29.3 %)
Portugal	18 (24 %)
Netherlands	15 (20 %)
Ireland	6 (8. %)
Czech Republic	5 (6.7 %)
United Kingdom	3 (4 %)
Italy	2 (2.7 %)
Austria	1 (1.3 %)
Belgium	1 (1.3 %)
Romania	1 (1.3 %)
Sweden	1(1.3 %)
**Age**	
Mean age (SD)	48.3 (11.0)
**Sex**	
Female (%)	51 (68 %)
Male (%)	23 (30.7 %)
Non-binary (%)	1 (1.3 %)
**Mean work experience in years (SD)**	11.5 (8.3)
**Teaching role**	
Educator	42 (56 %)
Course coordinator/developer and educator	28 (37.3 %)
Course coordinator/developer	5 (6.7 %)

### 3.2 Acceptability of using VR in serious illness communication courses

The overview of the ADOPT-VR acceptability outcomes is presented in Table 2. The highest acceptability outcomes were perceived usefulness of VR and attitude of the educators toward VR. At the same time, intention to use VR seems to be moderate, with a mean of 13.6. Notably, some dimensions such as self-efficacy (M = 4.9), time (M = 4.9) and perceived technical support (M = 3.6) scored relatively low. All scales of the ADOPT-VR questionnaire had a good or excellent reliability (Cronbach's alphas are presented in Table 2).

**Table 2:** The concepts measured by ADOPT-VR presented as means and standard deviations, and Cronbach's α.

Concept	Definition	Mean ± SD	Scale (α)
Attitude	Educators' general feelings about using VR technologies in their courses	20.4 ± 5.5	3–27 (0.93)
Perceived usefulness	Educators' beliefs that VR is a valuable education tool that will enhance the teaching process and will result in improved learning outcomes	21 ± 4.7	3–27 (0.87)
Perceived ease of use	Educators' beliefs that using VR in their teaching practice will be simple	14 ± 5.7	3–27 (0.83)
Compatibility	The extent to which educators believe that the use of VR fits with their current teaching approaches and meets their students' needs	10.4 ± 4.3	2–18 (0.80)
Social norms	Educators' beliefs about whether or not others think they should be using VR	20.3 ± 8.5	5–45 (0.84)
Behavioural control	Educators' perceptions of internal (e.g., knowledge and skills) and external factors (e.g., resources and supports) affecting their ability to use VR in their practice	7.8 ± 4.8	2–18 (0.86)
Self-efficacy	Educators' beliefs that they have the personal ability to use VR as an education tool with their students	4.9 ± 2.5	1–9 (−)
Time	Educators' belief that they have enough time to integrate VR in their teaching practice	4.9 ± 2.2	1–9 (−)
Technical support	Educators' belief that they have enough technical support available to integrate VR in their teaching practice	3.6 ± 2.4	1–9 (−)
Intention to use	Educators' intentions to use VR with their students in future	13.6 ± 5.4	3–27 (0.84)

### 3.3 Intention to use

When looking at the predictors of the intention to use VR in teaching, the overall model with nine predictors was significant (F(9, 65) = 17,26, R^2^ = 0.735, *p* < .001). Two of the concepts of the ADOPT-VR significantly positively predicted the intention to use: compatibility with the current teaching approaches (beta = 0.319, t(65) = 2.267, *p* = .027) and social norms (beta = 0.296, t(65) = 2.941, *p* = .005). The other predictors were not statistically significant (all p's > 0.05).

### 3.4 Barriers to use of VR in teaching

Barriers were perceived within several domains (see Table 3), of which ‘Resource and Knowledge’ and ‘Training’ constraints were most often mentioned and perceived as significant barriers. More specifically, the most mentioned barriers to using VR in teaching were the lack of financial support (62.7 %) and a lack of training opportunities related to VR (54.7 %), alongside teaching space issues (53.3 %). This overlaps with the most significant barrier for preventing the integrating VR in their teaching practice, which were the lack of training opportunities (22.6 %) and financial resources (17.2 %).

**Table 3:** The list of barriers to using VR.

Barrier	Mentioned as a barrier n (%⁎)	Mentioned as the most significant barrier n (%)
**Resource constraints**		
Lack of financial support⁎⁎	47 (62.7 %)	16 (17.2 %)
Lack of equipment⁎⁎⁎	–	12 (12.9 %)
Teaching space issues⁎⁎	40 (53.3 %)	4 (4.3 %)
Lack of technical support⁎⁎⁎	–	6 (6.5 %)
Lack of software (VR communication trainings) ^⁎⁎⁎^	–	3 (3.2 %)
**Knowledge and training constraints**		
Lack of VR knowledge/ training to use VR⁎⁎	41 (54.7 %)	21 (22.6 %)
Poor evidence to support the use of virtual reality⁎⁎	19 (25.3 %)	10 (10.8 %)
Lack of access to evidence on virtual reality’s effectiveness⁎⁎	31 (41.3 %)	0
**Organisational constraints**		
Lack of support from the management⁎⁎	26 (34.7 %)	1 (1 %)
Lack of acceptance from other educators⁎⁎⁎	–	1 (1 %)
Long implementation time of new technology in the institution⁎⁎⁎	–	1 (1 %)
**Time constraints**		
Lack of time to learn how to use the virtual reality system`	30 (40 %)	11 (11.8 %)
The time required to use virtual reality in a class⁎⁎	20 (26.7 %)	
**Student-related constraints**		
Poor motivation of students to participate⁎⁎	4 (5.3 %)	2 (2.2 %)
Lack of appropriate students with which to use VR	3 (4 %)	0
**Other**		
Absence of personal connection in VR necessary for teaching communication⁎⁎⁎	–	2 (2.2 %)
Data privacy concerns⁎⁎⁎	–	1 (1 %)
Lack of motivation	–	1 (1 %)

⁎ Percentage of participants who mentioned the barrier. 93 barriers were mentioned by 75 participants.

⁎⁎ The barrier chosen from the list of ADOPT-VR questionnaire.

⁎⁎⁎ The barriers mentioned by the participants in response to the open question about the most significant barrier.

### 3.5 Facilitators for using VR

When asked what would help them to incorporate VR into their teaching practice, the participants mentioned ‘Availability of training to use VR’ as the most common facilitator (22.0 %), followed by several facilitators from the domain ‘Resource availability’, namely the ‘Availability of technical support’ (11.0 %), ‘Availability of financial support’ (9.0 %), ‘Availability of equipment’ (9.0 %) and ‘Availability of software’ (9.0 %). The full list of facilitators is presented in Table 4.

**Table 4:** List of the facilitators for implementing VR in their teaching practice mentioned by the participants.

Facilitators	Frequency n (%⁎)
**Resource availability**	
Availability of technical support	11 (11 %)
Availability of financial support	9 (9 %)
Availability of equipment	9 (9 %)
Availability of software (VR communication trainings)	9 (9 %)
**Knowledge and training availability**	
Availability of training to use VR	22 (22 %)
Acquiring personal experience with VR	5 (5 %)
Evidence on virtual reality's effectiveness	9 (9 %)
Help from colleagues	5 (5 %)
Better understanding of the technology	1 (1 %)
**Organisational support**	
Support from the management	5 (5 %)
Availability of an implementation strategy	1 (1 %)
Support from national bodies	1 (1 %)
Support from course developers	1 (1 %)
**Student-related facilitators**	
Interest of students	2 (2 %)
**Time availability**	
Availability of time	5 (5 %)
**Other**	
Lower costs	2 (2 %)
Availability of ways to share materials	1 (1 %)
Addition to the traditional methods	1 (1 %)
Nothing	1 (1 %)

⁎ Percentage of participants that mentioned the facilitator. In total 100 facilitators were mentioned by 75 participants.

### 3.6 Advantages and disadvantages of using VR

A range of perceived benefits associated with the use of VR in teaching serious illness communication was identified. The most frequently cited benefit was immersion (17.5 %), possibility for repetition and practice (12.4 %) and cost and resource efficiency (12.4 %).

Analysis of the survey responses revealed a range of potential disadvantages of VR. The most frequently mentioned disadvantages were from the domain ‘Limitations in realistic learning experience’ and included the lack of human interaction (35.6 % of responses), limited realism (12.6 % of responses) and other learning experience issues (12.6 %). Technical issues were mentioned as a potential disadvantage by 9.2 % of participants. The full list of advantages and disadvantages reported by the participants is presented in Table 5.

**Table 5:** Frequencies of advantages and disadvantages of using VR mentioned by the participants and example quotes.

Category	Frequency n (%)	Example of a response
**Advantages**		
**Learning experience**		
*Immersion*	17 (17.5 %)	*“Greater immersion, which makes the situation more life-like”*
*Safe practice environment*	10 (10.3 %)	*“Safe to practice because there is not a real person that they can harm with their communication”*
*Engagement and motivation*	7 (7.2 %)	*“It could be a new refreshing way for students to put theory into practice, which could enhance their enthusiasm for learning new skills”*
*Improved learning outcomes*	4 (4.1 %)	*“VR may result in improved learning outcomes for my students”*
*Feedback and assessment*	1 (1 %)	*“Variation, training in specific techniques and feedback mechanisms”*
**Flexibility andStandardization**		
*Repetition and practice*	12 (12.4 %)	*“Students can have more practice time”*
*Flexibility*	8 (8.2 %)	*“Students can do this in their own time”*
*Variety of scenarios*	8 (8.2 %)	*“The variety in patients. Right now we use simulated patients to train skills. It would be amazing to have a more diverse pool in backgrounds.”*
*Supplement to traditional methods*	6 (6.2 %)	*“I think VR should only be seen as an addition but not as a replacement of face-to-face encounters”*
*Standardization*	4 (4.1 %)	*“I think it could be a standardised approach allowing the individuality of the situation unfold as per the students selections”*
**Technical and logistical benefits**		
*Accessibility*	4 (4.1 %)	*“The advantage is easier access, thus the easier planning and scheduling, reaching more students in case of lack of teachers”*
*Technological advancement*	4 (4.1 %)	*“New exciting method”*
**Cost and resource efficiency**		
*Cost and resource efficiency*	12 (12.4 %)	*“Not as time- and resource-consuming as a role play”*
**Disadvantages**		
**Limitations in realistic learning experience**		
Lack of human interaction	31 (35.6 %)	*“I fear it disconnects students more and more from real life interaction with physical human beings (the essence of life and the medical field)”*
Limited realism	11 (12.6 %)	*Real acting patients can seem more real to many students. They might confuse training with a video game.*
Other learning experience concerns	11 (12.6 %)	*Getting the level of the experience right for the student*
**Flexibility**		
Content and flexibility issues	8 (9.2 %)	*“I think that at this moment the VR is limited in terms of the content, variability and flexibility of the script”*
**Technical and logistical challenges**		
Practical and logistical challenges	9 (10.3 %)	*“Not all students will have the equal opportunity to engage - with 350 students in some cohorts this is not a practical approach”*
**Cost and resources**		
Costs	9 (10.3 %)	*“It is expensive and has to be maintained and regularly updated”*
Technical issues	8 (9.2 %)	*“The disadvantages seem to me to be mainly the practical matters,* i.e. *technology that is difficult or does not work”*

## 4 Discussion

### 4.1 Discussion and innovation

In this cross-sectional survey study, we investigated the acceptance of using VR training in classes on difficult conversations among health professions educators in Europe, including what predicted their intention to use VR. Additionally, we investigated facilitators and barriers, advantages and disadvantages of implementing VR into serious illness communication education. We found that participants had a moderate intention to use VR in their teaching practice with a large variability among participants. Both perceived barriers and facilitators were found at the level of funding and training to use VR. The realistic, immersive, and flexible nature of VR was mentioned as an important advantage, while a key disadvantage was the lack of human interaction in VR communication training.

In general, the score for the intention to use VR in teaching practice was moderate in our sample with a large variability. At the same time, participants had a positive attitude and high perceived usefulness of VR. However, when looking at the predictors of the intention to use VR, neither attitude, nor perceived usefulness significantly predicted the intention to use. Social norms and compatibility with teaching methods were the only two significant predictors of the intention to use VR. Both of these parameters were rated as moderate. These results are in line with previous research using the Decomposed Theory of Planned Behaviour in respect to adoption of new VR technology in clinical practice. These studies also found no link between all constructs of the theory with the intention to use the technology [30,31]. It is however worth considering that while these studies were conducted in clinical contexts, our study was focused on health professions educators. Possibly, if VR fits in the programs and norms of medical schools, educators might see it as more acceptable.

In general, participants mentioned more perceived barriers than facilitators for implementing VR in education. Almost half of participants named lack of financial support as a barrier. Educators were worried that implementing VR would incur significant costs. Financial obstacles are indeed one of the most commonly mentioned barriers for implementing new technology in education [[32], [33], [34]]. Paradoxically, when identifying potential advantages of implementing VR into their education, several participants mentioned that VR use could also decrease costs if it replaces the work with actors for roleplay simulation. Moreover, participants considered that VR might also decrease the load on the educators by allowing students to practise on their own. A study comparing the costs of an evacuation VR training with live exercises for hospital workers found that initially VR training is more expensive; however, when the training gets repeated, its development costs drop per participant, while the cost of live exercises remain fixed [35]. Similar research is needed in the domain of serious illness communication training to see whether the costs of implementing VR (e.g., hardware, software, and technical support) could be offset by the potential savings of using it repeatedly with a large number of students.

The second most listed perceived barrier was the lack of educational opportunities related to VR. A large number of participants perceived that they had insufficient knowledge of VR and of how to implement it to their practice. At the same time, the most mentioned perceived facilitator to using VR was the availability of training for educators. It has been previously shown that lack of experience with VR technology leads to more reservations about implementing it to medical education [36]. To successfully implement VR, it is essential to provide targeted training for educators, equipping them with the necessary knowledge and skills to effectively integrate the technology into their teaching practices. A “train-the-trainers” approach, supported by comprehensive manuals and instructional materials, could serve as a valuable strategy to enhance educators' confidence and competence in utilizing VR-based training tools.

The perceived disadvantage of a lack of human interaction and authenticity when training communication skills with VR scenarios touches upon the fundamental question of whether VR can be used to properly train and prepare students for complex and nuanced interactions in serious illness communication in practice. In these interactions, clinicians not only need to be able to provide information in a clear manner while expressing verbal and non-verbal empathy, but also be able to foster human connection. Connecting to a patient story [37] and picking up on subtleties of spoken and unspoken communication (van Vliet et al., unpublished results), often in the setting of a long-term clinician-patient relationship is paramount. Interestingly, a recent study showed that VR training improved clinicians' ability to demonstrate empathy more than traditional role-played training [26]. Therefore, one could expect immersive realities, such as VR, to have the potential to enrich empathic sensitivity and competencies among future healthcare professionals as these innovative tools offer immersive experiences that nurture and cultivate ethical, compassionate, and empathetic decision-making skills [38]. More research is needed into the ability of VR to teach the nuances of serious illness communication in real settings.

Participants listed a number of potential advantages of VR training including: immersiveness of the training that can motivate the students, opportunities for repetition and practise that it would provide and possibility to practise in a safe environment. These benefits of the VR were also mentioned in a previous study on the feasibility of a VR communication training for dietitians [39]. Several participants mentioned that they would like to use VR as a supplement to their standard teaching methods but did not necessarily want to completely replace practise with real humans with VR, which can be a solution addressing the mentioned concerns of the participants and at the same time leveraging the advantages of VR.

Next to the importance of tackling the perceived barriers and disadvantages of VR by creating realistic cost-effective VR communication training including implementation guidelines, it is equally important – as also mentioned by participants in our study – to build on the evidence of VR communication training. While doing so the researchers will face the same difficulties more traditional trainings are facing; while it might be relatively easy to establish whether a training improves clinicians' communication skills in avatar/role-played scenarios [26,40] or even real interactions [41], effects on more down-stream outcomes such as improved health/treatment understanding/awareness related to health by patients are less straightforward [42]. Ultimately, providing educators with evidence-based VR training, will be crucial for the successful implementation of VR-based training alongside face-to-face training opportunities.

This study has several notable strengths, including its novel exploration of health professions educators' views on using VR for serious illness communication training, an area that has not yet been widely investigated. The international range of participants, representing various European countries, adds to the study's relevance and generalizability within high-income settings. Furthermore, the innovative potential of VR in health professions education, as highlighted by the participants, points to promising directions for integrating immersive technologies into training programs.

Our study is not without limitations. The sample size was relatively small, and the recruitment strategy—largely through word of mouth and social media—did not allow us to calculate an exact response rate and a potential for the selection bias exists. Additionally, the study included only participants from high-income European countries, with some countries being overrepresented, limiting the broader generalisability of findings. Another limitation is that the interpretation of the intention to use VR scores was based on our defined thresholds, rather than established guidelines from the literature. Also, we did not assess the level of experience of the students (Bachelor, Master, Specialisation), which might influence the views on VR of their teachers. Finally, while the study offers initial insights, the cross-sectional design prevents drawing conclusions about changes in attitudes over time.

### 4.2 Conclusion

In conclusion, while educators perceive that VR offers promising advantages such as immersion and flexibility, addressing technical and financial barriers in a systematic manner is essential to encourage the broader adoption of VR in medical education. VR could serve as a valuable supplement to traditional teaching methods, but further research is necessary to build strong evidence base and ensure that it effectively enhances or complements face-to-face training communication in serious illness. Such research ought to explore the usability, feasibility, acceptability, and potential effectiveness of bespoke VR scenarios used to teach difficult communication.

## CRediT authorship contribution statement

**Aleksandrina Skvortsova:** Writing – original draft, Data curation, Methodology, Conceptualization, Writing – review & editing, Formal analysis. **Stephanie Stiel:** Funding acquisition, Conceptualization, Writing – review & editing. **Kambiz Afshar:** Funding acquisition, Conceptualization, Writing – review & editing. **Hanna A.A. Röwer:** Writing – review & editing, Conceptualization. **Claudia Bausewein:** Writing – review & editing, Funding acquisition, Conceptualization. **Irene Hartigan:** Writing – review & editing, Funding acquisition, Conceptualization. **Mohamad M. Saab:** Writing – review & editing, Funding acquisition, Conceptualization. **Sandra Martins Pereira:** Writing – review & editing, Funding acquisition, Conceptualization. **Pablo Hernández-Marrero:** Writing – review & editing, Funding acquisition, Conceptualization. **Jan Hrdlička:** Writing – review & editing, Funding acquisition, Conceptualization. **Jiri Wild:** Writing – review & editing, Funding acquisition, Conceptualization. **Kateřina Rusinová:** Writing – review & editing, Funding acquisition, Conceptualization. **Martin Loučka:** Writing – review & editing, Funding acquisition, Conceptualization. **Lucie Hrdličková:** Writing – review & editing, Funding acquisition, Conceptualization. **Martin Zielina:** Writing – review & editing, Funding acquisition, Conceptualization. **Cathy Payne:** Writing – review & editing, Funding acquisition, Conceptualization. **Liesbeth M. Van Vliet:** Writing – original draft, Funding acquisition, Supervision, Formal analysis, Methodology, Conceptualization.

## Funding

This study was funded by ERASMUS PLUS awarded to consortium VR-TALKS.

Sandra Martins Pereira is Principal Investigator funded by the Portuguese Foundation for Science and Technology (FCT) under the Scientific Employment Stimulus (CEECINST/00137/2018, DOI 10.54499/CEECINST/00137/2018/CP1520/CT0010) at CEGE: Research Centre in Management and Economics, Ethics and Sustainability Research Area.

## Declaration of competing interest

The authors originating from ComGuide (Jan Hrdlička, Lucie Hrdličková and Jiri Wild) declare a potential conflict of interest regarding the involvement of them in the commercialization of VR. While these authors do not directly generate revenue from the research done in this project, they are involved in marketing of VR products. There is a possibility that the project activities could indirectly benefit the authors' commercial interests by increasing visibility, market perception, or future sales prospects of these VR products. However, measures have been implemented to ensure that all project activities are conducted transparently, objectively, and in alignment with the agreed-upon research objectives. The partner has committed to separating their role in the project from their commercial activities and will not exert influence over project decisions that are not directly related to the project's success. All stakeholders will be informed regularly of any changes that could potentially impact this disclosed conflict of interest.

The other authors declare that they have no known competing financial interests or personal relationships that could have appeared to influence the work reported in this paper.
